# Supplementary material for: Enhanced recombinant protein production in CHO cell continuous cultures under growth-inhibiting conditions is associated with an arrested cell cycle in G1/G0 phase
Source: PLoS One. 2022 Nov 14;17(11):e0277620. doi: 10.1371/journal.pone.0277620 (PMC9662745; doi:10.1371/journal.pone.0277620)
Supplement: S2 File — (PDF) [file pone.0277620.s002.pdf]

Supplementary file

Enhanced recombinant protein production in CHO cell continuous cultures under growth-inhibiting conditions is associated with an arrested cell cycle in G1/G0 phase.

Verónica Avello[1,2 #a], Mauro Torres[3,4], Mauricio Vergara[1], Julio Berrios[1], Norma A. Valdez-Cruz[5], Cristian Acevedo[2,6,7], Maria Molina Sampayo[9], Alan Dickson[3,4] and Claudia Altamirano[3,8,*]

[1] Escuela de Ingeniería Bioquímica, Pontificia Universidad Católica de Valparaíso, Av. Brasil 2085, P.O. Box Valparaíso, Chile

[2] Centro de Biotecnología, Universidad Técnica Federico Santa María, Valparaíso 2390123, Chile

[3] Manchester Institute of Biotechnology, Faculty of Science and Engineering, University of Manchester, Manchester, UK.

[4] Department of Chemical Engineering, Biochemical and Bioprocess Engineering Group, University of Manchester, Manchester, UK.

[5] Departamento de Biología Molecular y Biotecnología, Instituto de Investigaciones Biomédicas, Universidad Nacional Autónoma de México, Ciudad de México, México

[6] Centro Científico Tecnológico de Valparaíso CCTVaL, Universidad Técnica Federico Santa María, Valparaíso 2390123, Chile

[7] Departamento de Física, Universidad Técnica Federico Santa María, Valparaíso 2390123, Chile

[8] Centro de InmunoBiotecnología, Universidad de Chile, Santiago, Chile.

[9] Centro Regional de Estudio en Alimentos Saludables, R17A10001, Av. Universidad 330, Valparaíso, Chile

[#a] Present Address: Biotechnology and Biopharmaceutical Laboratory, Pathophysiology Department, School of Biological Sciences, Universidad de Concepción, Victor Lamas 1290, P.O. Box 160-C, Concepción, Chile Curauma, Valparaíso 2373223, Chile

Correspondence: Prof. Claudia Altamirano, School of Biochemical Engineering, Pontificia Universidad Católica de Valparaíso, Brasil Avenue 2085, Valparaíso, Chile

E-mail: claudia.altamirano@pucv.cl

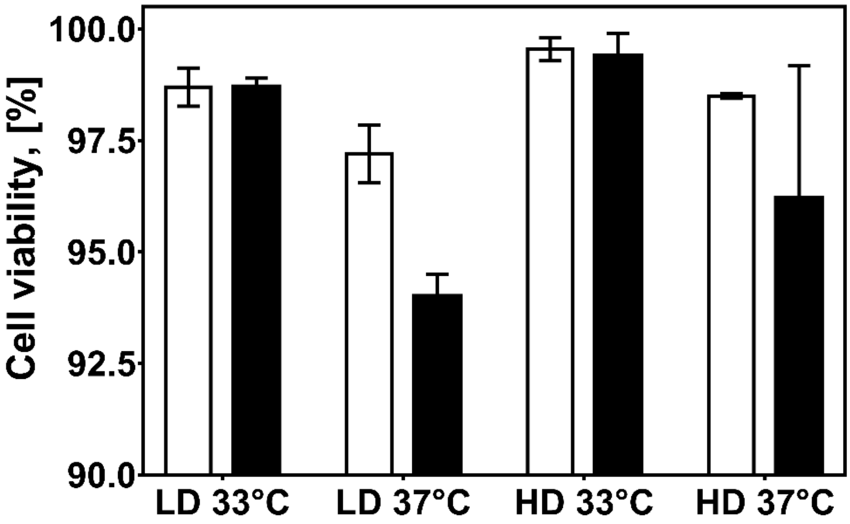

S1 Fig. Cell viability profiles of CHO cells in continuous cultures. (*white*) without NaBu and (*black*) with NaBu. Experimental values represent the mean of two biological replicates ± SEM.

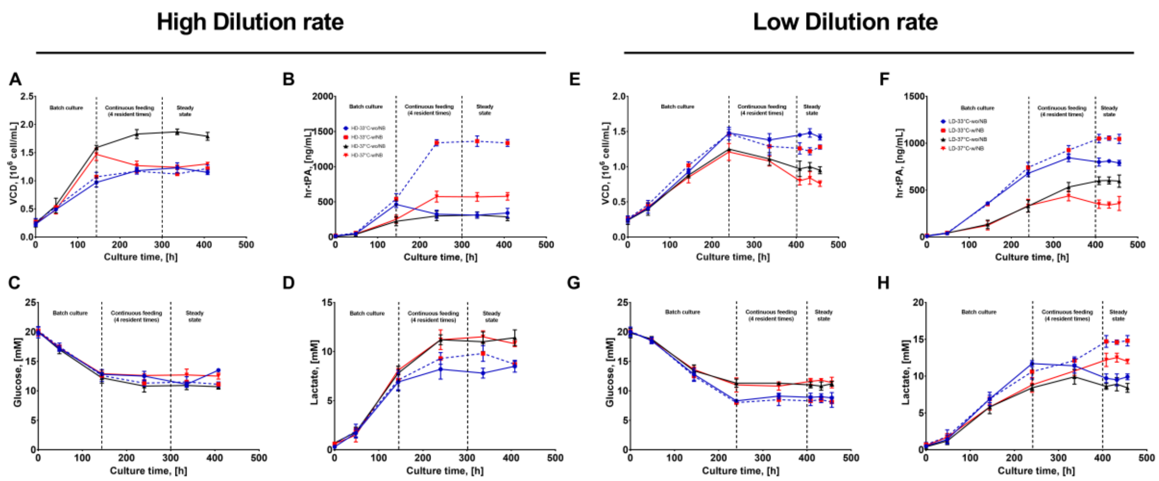

S2 Fig. Profiles of CHO cell performance in chemostat cultures at steady state. (*black*) Control conditions (HD-37°C-wo/NB and LD-37°C-wo/NB); (*blue*) Temperature effect (HD-33°C-wo/NB and LD-33°C-wo/NB); (*red*) Sodium butyrate effect (NB) (HD-37°C-w/NB and LD-37°C-w/NB); (*red square and blue line*) Combined effect of temperature and NB) (HD-33°C-w/NB and LD-33°C-w/NB). A) and E) Viable cell densities (VCDs). B) and F) hr-tPA production. C) and G) Glucose concentration in medium. D) and H) Lactate concentration in medium. Cell viability remained above 95% for all cultures (**Fig S1**). Experimental values represent the mean of two biological replicates ± SEM.

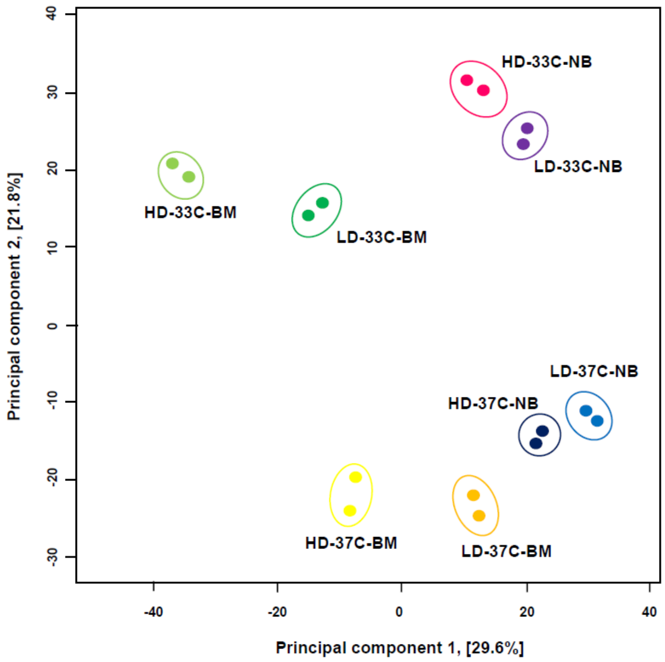

S3 Fig. Principal component analysis of transcriptome data of CHO cells in continuous cultures. The figure illustrates the position of observations in the ordination where specific culture conditions are represented.
