# Supplementary material for: Enhanced recombinant protein production in CHO cell continuous cultures under growth-inhibiting conditions is associated with an arrested cell cycle in G1/G0 phase
Source: PLoS One. 2022 Nov 14;17(11):e0277620. doi: 10.1371/journal.pone.0277620 (PMC9662745; doi:10.1371/journal.pone.0277620)
Supplement: Attachment — Submitted filename: Response to reviewers.docx [file pone.0277620.s003.docx]

Ioscani Jimenez del Val, Ph.D.

Academic Editor

PLOS ONE

September 2022

Dear Editor Jimenez del Val,

Thank you for your communication regarding our manuscript entitled “**Enhanced recombinant protein production in CHO cell continuous cultures under growth-inhibiting conditions is associated with an arrested cell cycle in G1/G0 phase**" (PONE-D-22-10823). It is with pleasure that I resubmit a revised version of manuscript for consideration by *PLOS ONE*.

I would like to thank you and the reviewers for the time invested in review our manuscript, as well as the positive and constructive comments. I hope the revised version of the manuscript will be considered suitable for publication by *PLOS ONE.*

In the revised version of the manuscript, we have included the profile data of chemostat cultures to confirm the steady state operation. This data includes cell growth, hr-tPA production and metabolic profiles of glucose and lactate during the entire culture, and presented in S2 Fig in the Supplementary file. We have also modified the original manuscript format to adjust PLOS ONE’s style requirements.

Below, we have given our response to each specific comment and suggestion, listing them as described in your communication to us. Changes in the revised manuscript are highlighted in red.

Sincerely,

Claudia Altamirano, PhD

Professor of Biochemical Engineering

Director, ANILLO ACT 210068 Research in Science and Technology

Principal Investigator, Center of Interventional Medicine for Precision and Advanced Cellular Therapy

School of Biochemical Engineering

Faculty of Engineering

Pontifical Catholic University of Valparaíso

2085 Brasil Avenue

Valparaíso

Chile

**Editor comments:**

“Based on reviewer comments, I recommend that the authors consider including time profiles for the continuous cultures performed under different treatments (mild hypothermia, NaBu, and control). This will allow the readers to confirm that steady states were indeed achieved and will strengthen the conclusions presented by the authors. It may be as simple as adding a supplementary file with the data and referencing it in the manuscript. Once data confirming steady state during continuous cultures is made available, I the manuscript will be ready for publication in PLoS One.”

**A:** We have included the data regarding our chemostat cultures during the steady state as requested. This data was included in the supplementary file and mentioned in the manuscript to provide the readers an understanding how cells behaved during the continuous cultivation.

**Reviewer #1**:

General comments:

“*This paper focused on the gene expression under continuous CHO cultivation. It seems that this paper contains valuable information. I think that the manuscript should be accepted with major revision. Chemostat data should be shown in the manuscript. Authors mentioned that all chemostat cultures were operated at a steady-state. All analytical data were analyzed on the basis of chemostat steady-state. Without this information, it is very difficult to elucidate the obtained data. However, detail time course of cell and limiting-substrate concentrations were not shown. To confirm chemostat steady-state, the detail time course should be shown in the revised manuscript*”.

**A:** Thank you for the time invested in the review of this manuscript and for the positive comments. We have included the data that supported the achievement of a steady state in our chemostat cultures. This data includes profiles of cell growth, hr-tPA production, glucose and lactate throughout the cultures. We included this data in the Supplementary file as S2 Fig, and mentioned in the main manuscript.

**Reviewer #2**:

General comments:

“*This is a good paper that examines the mechanism of the effect of low temperature and NaBu, which is considered effective in a production system using CHO cells. It is especially excellent that it has been validated in CHEMOSTAT culture*”.

**A:** We thank the reviewer for the time taken to review this paper and for the positive comments.
