# Supplementary material for: Enhanced recombinant protein production in CHO cell continuous cultures under growth-inhibiting conditions is associated with an arrested cell cycle in G1/G0 phase
Source: PLoS One. 2022 Nov 14;17(11):e0277620. doi: 10.1371/journal.pone.0277620 (PMC9662745; doi:10.1371/journal.pone.0277620)
Supplement: Attachment — Submitted filename: Response to reviewers.docx [file pone.0277620.s004.docx]

Ioscani Jimenez del Val, Ph.D.

Academic Editor

PLOS ONE

October 2022

Dear Editor Jimenez del Val,

Thank you for your communication regarding our manuscript entitled “**Enhanced recombinant protein production in CHO cell continuous cultures under growth-inhibiting conditions is associated with an arrested cell cycle in G1/G0 phase**" (PONE-D-22-10823). It is with pleasure that I resubmit a revised version of manuscript for consideration by *PLOS ONE*.

I would like to thank you and the reviewers for the time invested in review our manuscript, as well as the positive and constructive comments. I hope the revised version of the manuscript will be considered suitable for publication by *PLOS ONE.*

In the revised version of the manuscript, we have addressed the concerns about the achievement of a steady state in the chemostat cultures. Specifically, we have modified Fig S2 to separate the data regarding the two-dilution factor conditions, so the data at steady state can be seen more clear compared to the previous format. We also discussed the adaptation process that cells undergo during the feeding regime until they reach steady state conditions.

Below, we have given our response to each specific comment and suggestion, listing them as described in your communication to us. Changes in the revised manuscript are highlighted in red.

Sincerely,

Claudia Altamirano, PhD

Professor of Biochemical Engineering

Director, ANILLO ACT 210068 Research in Science and Technology

Principal Investigator, Center of Interventional Medicine for Precision and Advanced Cellular Therapy

School of Biochemical Engineering

Faculty of Engineering

Pontifical Catholic University of Valparaíso

2085 Brasil Avenue

Valparaíso

Chile

**Editor comments:**

*The authors have successfully addressed all comments made by the reviewers. However, additional questions have been raised regarding the existence of a true steady state during the continuous cultures. As suggested by Reviewer 1, minor revisions should be included in the manuscript to briefly discuss deviations from steady state and potential implications on the broader conclusions (i.e., that cell cycle arrest underlies increased productivity) presented in the manuscript. With these minor revisions, the manuscript will be ready for publication in PLoS One and will be of high interest to the journal's readership and the broader CHO community.*

**A:** Thank you for the time taken for reviewing this paper and the positive comments. We addressed the concerns of Reviewer #1 in the section below.

**Reviewer #1:**

**General comment:**

*This paper focused on the gene expression under continuous CHO cultivation. It seems that this paper contains valuable information. I think that the manuscript should be accepted with minor revision. Steady-state is not clear. According to time course of chemostat culture as shown in S2 Fig., I think that it is not steady-state. For example, VCD, lactate concentrations, other concentrations are not constant. Also, viability data is necessary. Viability (Nt/Nv) should be constant for steady-state. Limiting-substrate is not clear. According to the difference of limiting substrate, the steady-state should be changed (i.e.,doi: 10.1007/s00449-009-0351-8). Authors should clearly mention it and discuss about it in the revised manuscript.*

**A:** We agree with the reviewer that Fig S2 did not show clearly the steady state, although we believe that this issue was associated with the number of conditions plotted in the graphs that made it difficult to identify the steady state from the adaptation process to the feeding regime. To address this point, we have modified Fig S2 (see below new figure) to show the initiation of the feeding regime, the corresponding adaptation process and the steady state. Cell viability was above 95% for all cultures and showed in FigS1 for the steady state.

We also agree with the reviewer and the editor that the slight variations during the steady state, particularly for glucose and lactate in low temperature at high dilution rate should be discussed. We have addressed this point in the discussion section.

Discussion section (Lines 441-457, Pages 13-14):

“*This study has provided novel insights into the regulation of the cell cycle and its relationship to the productive phenotypes of CHO cells. Nonetheless, these results must be interpreted with caution, and potential limitations should be borne in mind. An initial limitation concerns the use of chemostat cultures, a cultivation method that does not represent the reality of current industrially-relevant manufacturing settings, where cells can reach high cell density and product yields. However, continuous culture represents an excellent tool for investigating the isolated effects of environmental parameters on cell physiology and performance. Additionally, findings made in continuous cultures have been shown to be relevant in other cultivation systems [25,43,44]. Another important consideration of using continuous culture is the cellular adaptation to the feeding regime. This adaptation often leads to changes in cell density, r-protein production and metabolism during the initial residence times (time when medium is renewed in the vessel) (****Fig S2****). In our experimental design, we consider that steady state was attained when the concentration of viable cells and metabolites are in a 5% deviation range between consecutive points. Our data showed that four residence times enabled the achievement of steady state conditions. Whilst we found small variation in glucose and lactate at HD-33°C conditions (despite the constant cell density and hr-tPA production), these slight deviations did not disrupt the steady state conditions and the biological effects of both low temperature and sodium butyrate observed in this study.”*

**New Supplementary Figure S2**

**S2 Fig. Profiles of CHO cell performance in chemostat cultures at steady state.** (*black*) Control conditions (HD-37°C-wo/NB and LD-37°C-wo/NB); (*blue*) Temperature effect (HD-33°C-wo/NB and LD-33°C-wo/NB); (*red*) Sodium butyrate effect (NB) (HD-37°C-w/NB and LD-37°C-w/NB); (*red square and blue line*) Combined effect of temperature and NB ) (HD-33°C-w/NB and LD-33°C-w/NB). A) and E) Viable cell densities (VCDs). B) and F) hr-tPA production. C) and G) Glucose concentration in medium. D) and H) Lactate concentration in medium. Cell viability remained above 95% for all cultures (**Fig S1**). Experimental values represent the mean of two biological replicates ± SEM.

**Previous Supplementary Figure S2**
